# Acetylcholinesterase secreted by *Anisakis simplex* larvae (Nematoda: Anisakidae) parasitizing herring, *Clupea harengus*: an inverse relationship of enzyme activity in the host–parasite system

**DOI:** 10.1007/s00436-014-3878-9

**Published:** 2014-04-05

**Authors:** Magdalena Podolska, Katarzyna Nadolna

**Affiliations:** National Marine Fisheries Research Institute, Kollataja 1, 81-332 Gdynia, Poland

**Keywords:** *Anisakis simplex*, Herring, Acetylcholinesterase, Host–parasite system

## Abstract

Acetylcholinesterase (AChE) is a key enzyme involved in nerve impulse transmission in both vertebrates and invertebrates. In addition to neuromuscular AChE, many parasitic nematodes synthesize AChE in secretory glands and release the enzyme into their external environment. In this study, we evaluate the activities of both somatic and secreted AChE from larvae (L3) of the parasitic nematode *Anisakis simplex*, and compare these to the AChE activity in its host, herring, *Clupea harengus. A. simplex* larvae were obtained from a herring sampled in three areas of the southern Baltic. Enzyme kinetics were determined for excretory/secretory (E/S) products and somatic extracts of larvae as well as for herring muscle tissue. The results reveal that mean AChE activity is approximately fourfold higher in E/S products and eightfold higher in somatic extracts of post-secretory *A. simplex* larvae than in host muscle tissue. The level of AChE activity in nematodes is inversely related to the enzyme activity in their hosts, i.e. reduced AChE activity in herring was accompanied by increased enzyme activity in its parasites. The physiological function of AChE secreted by parasitic nematodes has been widely discussed in the literature, and numerous roles for this form of enzyme have been suggested. The results of our investigation indicate that AChE secretion by *A. simplex* larvae may constitute an adaptive mechanism that promotes survival under adverse environmental conditions. Larvae probably increase secretion of AChE in response to a direct and/or indirect effect of neurotoxic compounds. This is the first report of such a phenomenon in *A. simplex.*

## Introduction

Cholinesterases are polymorphic enzymes involved in nerve impulse transmission in both vertebrates and invertebrates. Two major classes of cholinesterase exist in vertebrates: acetylcholinesterase (AChE), whose preferred substrate is acetylcholine (ACh), and the closely related butyrylcholinesterase, which has a high affinity for butyrylcholine as a substrate (Massoulie 2002). In all invertebrates studied so far (i.e. nematodes and arthropods), the only cholinesterases found are of the AChE class (Toutant 1989; Combes et al. 2000; Kang et al. 2011a, b). Two different AChE genes have been defined in various arthropod species (Baxter and Barker 2002; Weill et al. 2002, 2003; Lee et al. 2006). Multiple forms of enzyme were identified in plant–parasitic nematodes: for example, three AChEs were defined in *Heterodera glycines* (Chang and Opperman 1992) and five in *Meloidogyne incognita* and *Meloidogyne arenaria* (Chang and Opperman 1991). Kang et al. (2011a) found three AChEs in pinewood nematode *Bursaphelenchus xylophilus*, each with a distinct physiological function.

Invertebrate AChE exists in both membrane-bound (amphiphilic) and soluble (hydrophilic) forms (Hyne and Maher 2003). Thus, membrane-bound (neuromuscular) AChEs have been characterized in parasitic nematodes such as *Trichinella spiralis* (DeVos and Dick 1992), *Parascaris equorum* (Talesa et al. 1997), *Nippostrongylus brasiliensis* (Hussein et al. 1999a) and *Dictyocaulus viviparus* (Lazari et al. 2004; Pezzementi et al. 2012). In the insect parasite *Steinernema carpocapsae*, two types of amphiphilic AChE were described by Arpagaus et al. (1992).

In addition to producing membrane-bound AChE, several species of parasitic nematodes secrete a soluble form of this enzyme into their external environment. This was first documented by analysis of secretory products obtained from cultures of *N. brasiliensis* (Sanderson 1972). Later studies on this nematode revealed three secreted forms of AChE (Grigg et al. 1997; Hussein et al. 1999b). Secretory AChE was also demonstrated in *Trichostrongylus colubriformis* (Griffiths and Pritchard 1994) and *Necator americanus* (Pritchard et al. 1994). Two variants of secretory AChEs were identified in *Setaria cervi* microfilariae (Sharma et al. 1998) and the lungworm *D. viviparus* (Lazari et al. 2003; Pezzementi et al. 2012). Recent results obtained by Podolska et al. (2012) suggest that *Anisakis simplex* larvae contain two AChE forms, which are proposed to be neuromuscular and secreted variants.

AChE is the biochemical target of organophosphates (OPs) and carbamate pesticides (CBs), irreversible or quasi-irreversible inhibitors of the enzyme, that are widely used to control insects. In addition to pesticides, other neurotoxic agents possess anti-AChE properties, including heavy metals, some types of chemical weapons and anthelmintic drugs, as well as cyanobacterial toxins. The application of pesticides can have unfortunate consequences, which are not limited to rural areas. Pesticides run off from fields to rivers, lakes and seas, reaching areas far from their point of use. For example, one side effect of the application of anticholinergic insecticides is the negative impact on the aquatic environment, exemplified by both acute and chronic toxicity in living organisms.

It is difficult to directly link AChE inhibition to OP or CB exposure, because these compounds have a short half-life. Most OPs degrade rapidly in the environment, and their concentrations in environmental samples may fall below detectable levels within hours to days (Fulton and Key 2001). The half-life of carbofurans is about 30–120 days, depending on the pesticide and on the pH and temperature of the aqueous environment (Morales et al. 2012).

AChE activity measurement is widely used in biomonitoring to assess neurotoxic effects in various marine species, including fish (Dembele et al. 1999; Niemi et al. 2002; Napierska and Podolska 2005; Barsiene et al. 2006). AChE is a very sensitive biomarker, and detection of inhibitory effects is possible even after exposure to low concentrations of insecticides (Habig et al. 1986). Although neurotoxic agents usually inhibit AChE activity in many species of fish, the opposite response may be observed in their parasites. Studies carried out by Podolska and Napierska (2006) revealed that AChE inhibition in muscle tissue of herring *Clupea harengus* caught in polluted areas was accompanied by very high enzyme activity in tissue extracts of herring parasites, *A. simplex* larvae. Further investigation (Podolska et al. 2008) indicated that AChE activity may increase in *A. simplex* larvae after experimental exposure to carbofuran. The most recent results show that AChE secreted by *A. simplex* larvae is characterized by a high level of activity (Podolska et al. 2012).

The objective of the study was to test the hypothesis that AChE secreted by *A. simplex* larvae from herring determines the relationship observed between host and parasite AChE activity in areas of the Baltic polluted with neurotoxic compounds.

## Materials and methods

### Sample collection

**Fig. 1:**
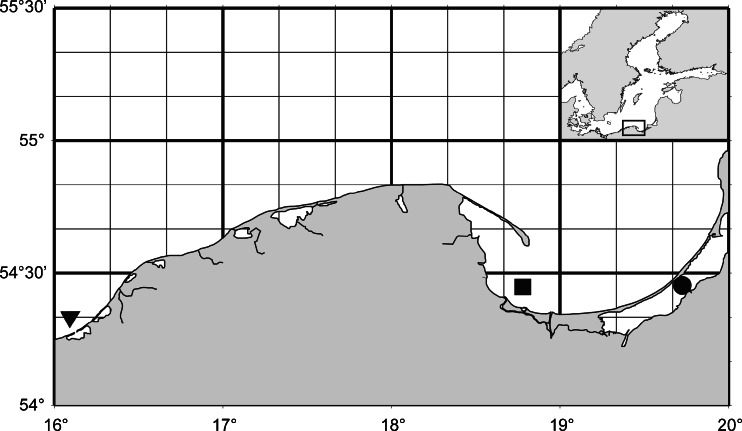
Sampling sites of herring: Vistula Lagoon (*black circle*), Gulf of Gdansk (*black square*), and middle coast (*black triangle*)

Samples of herring (*C. harengus*) were collected during the spring spawning period (from April to May 2011) in the coastal waters of the southern Baltic Sea in three locations: (1) Vistula Lagoon, (2) Gulf of Gdansk, and (3) the middle coast (Fig. 1). Standard measurements were performed on each fish. The presence of *A. simplex* larvae in the body cavity of each fish was recorded by macroscopic examination, and only fish containing parasites (total number 107) were chosen for further analyses. These individuals were placed with their anterior part facing leftwards, and a sample of muscle tissue was dissected from the posterior part of the fish, close to the tail fin. Samples of herring muscle were frozen immediately at −80 °C for further biochemical analyses. Live *A. simplex* larvae (L3) were collected, and the following procedure was used to acquire the excretory/secretory (E/S) products of the larvae: live nematodes (8–10 individuals per sample) were washed in physiological NaCl solution and maintained for 24 h at 4 °C in Eppendorf tubes containing 100 μl of physiological NaCl solution. The larvae were then removed from the tubes, washed and placed in new tubes. The E/S products and post-secretory larvae were frozen at −80 °C for further analyses.

### Preparation of tissue homogenates

Herring muscle tissue samples and *A. simplex* post-secretory larvae (pooled samples of 8–10 individuals) were homogenized using a mechanical blender. The AChE extraction was performed using 250–300 mg of herring muscle tissue (or 20–30 mg of larval tissue) in 0.02 M phosphate buffer (pH 7.0) containing 0.1 % Triton X-100. The herring tissue was homogenized in four volumes of buffer: 4 ml buffer per 1 g tissue wet weight (*A. simplex* larvae in 10 volumes of buffer, 10 ml buffer per 1 g tissue wet weight) and centrifuged at 10,000×*g* for 20 min at 4 °C. An aliquot of the supernatant (the “S9” fraction) was stored at −80 °C and used in the assay.

### Enzyme activity determination

The AChE activity determinations in herring and parasites (extracts of post-secretory larvae and E/S products obtained from live worms) were performed using a method described by Ellman et al. (1961) and adapted for use with a microplate reader (Bocquene and Galgani 1998). The enzyme kinetics were monitored at 412 nm using an absorbance microplate reader (iMark, Bio-Rad) and a standard reaction mixture (final volume 0.38 ml) containing 0.02 M phosphate buffer (pH 7.0), 0.01 M DTNB [5,5′-dithiobis(2-nitrobenzoic acid)] and 2.6 mM acetylthiocholine iodide (ACTC). Protein concentration was determined as described by Bradford (1976) using the Protein Kit II (Bio-Rad) and bovine serum albumin as the protein standard.

### Statistical analysis

Generalized linear models (GLMs) (McCullagh and Nelder 1989) were applied to analyze AChE activity in host and parasites with respect to the dependency on biological and spatial parameters. The following model was fitted (separately for host and parasites):

$$ \ln \left(\mathrm{AChE}\right)=\mathrm{area}+\mathrm{sex}+\mathrm{gon}+\mathrm{TL}+\mathrm{error} $$

where ln(AChE) represents log-transformed AChE activity of host or parasite; area, the sampling area; sex, the sex of the host; gon, the gonad developmental stage of the host; and TL, the total body length of the host. TL was taken as a covariate, whereas area, sex and gon were treated as factors. The error distribution was assumed to be normal, and the identity link function was used. Corner point parameterization was used, i.e. factor effects for level one were assumed to be zero for all factors. Thus, the factor effects for the other levels may be regarded as the difference between the effect at any given level and the effect at level one. First, the initial model (which included all variables and factors considered) was fitted. The significance of the factors and covariates was then tested, and only significant terms were left in the final model. Tests were performed by deletion, and those terms whose deletion did not result in a significant increase in deviance (i.e. the GLM measure of discrepancy between the modelled and observed values) were excluded from the model. Distributions of the model residuals were analyzed to test the model assumptions and performance.

## Results

**Fig. 2:**
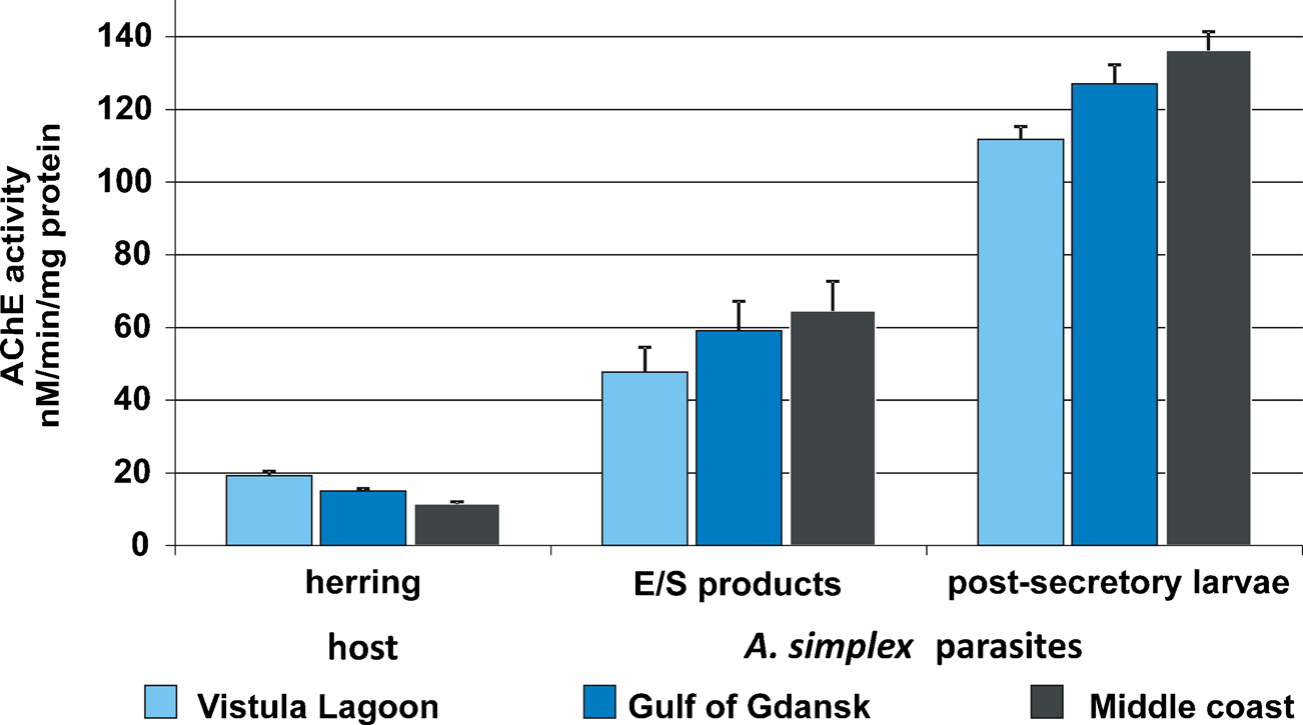
Acetylcholinesterase activity (nM/min/mg protein; with SE) in herring muscle tissue (host), E/S products and post-secretory larvae of *A. simplex* parasites in three areas of the Baltic Sea

The collected material comprised 107 sets of host and parasite samples (herring muscle tissue, *A. simplex* post-secretory larvae and its E/S products). Results of enzyme activity measurements indicated that the AChE level was much higher in *A. simplex* larvae than in herring host tissue and that an inverse relationship existed between enzyme activities of the host and its parasites (“mirror effect”). In the Vistula Lagoon samples, the highest mean activity in herring (19.32 nM/min/mg protein) corresponded to the lowest mean enzyme activity in *A. simplex* post-secretory larvae (111.78 nM/min/mg protein) and in E/S products obtained from parasites (47.73 nM/min/mg protein). Conversely, the lowest AChE activity in fish tissue (11.54 nM/min/mg protein) was associated with the highest enzyme activity in post-secretory larvae (136.26 nM/min/mg protein) and its E/S products (64.61 nM/min/mg protein; Fig. 2). On average, AChE activity was approximately fourfold higher in E/S products and eightfold higher in post-secretory larvae than in herring muscle tissue.

Mean enzyme activity was higher in female (15.93 nM/min/mg protein) than in male hosts (14.18 nM/min/mg protein), while values recorded for *A. simplex* were lower in parasites from females (121.36 nM/min/mg protein) than from males (130.74 nM/min/mg protein; Table 1). The highest enzyme activity in female herring sampled in the Vistula Lagoon (20.70 nM/min/mg protein) was accompanied by the lowest level of AChE in both post-secretory *A. simplex* larvae (107.61 nM/min/mg protein) and its E/S products (45.01 nM/min/mg protein; Table 1). The greatest divergence between male hosts (lowest level of 11.05 nM/min/mg protein) and post-secretory larvae (highest level of 138.75 nM/min/mg protein) was recorded in the middle coast (Table 1).

**Table 1:** Acetylcholinesterase activity (nM/min/mg protein; with SE) in herring muscle tissue (host) and *A. simplex* parasites (E/S products and post-secretory larvae) in three areas of the Baltic Sea, in relation to the host’s sex

Host’s sex	Area	*N*	Herring		*A. simplex*			
					E/S products		Post-secretory larvae	
Males	Vistula Lagoon	14	17.83	(1.61)	50.65	(10.15)	116.24	(4.62)
	Gulf of Gdansk	22	14.13	(0.92)	49.16	(9.20)	134.14	(7.16)
	Middle coast	16	11.05	(0.74)	56.65	(8.91)	138.75	(7.99)
Sum		52	14.18	(0.71)	51.87	(5.40)	130.74	(4.22)
Females	Vistula Lagoon	15	20.70	(1.75)	45.01	(9.48)	107.61	(5.24)
	Gulf of Gdansk	21	16.14	(1.29)	69.54	(12.31)	119.60	(6.49)
	Middle coast	19	11.95	(0.87)	71.31	(13.09)	134.16	(6.90)
Sum		55	15.93	(0.87)	63.46	(7.06)	121.36	(3.92)
Total Sum		107	15.08	(0.57)	57.83	(4.50)	125.92	(2.90)

*N* number of sample sets

**Fig. 3:**
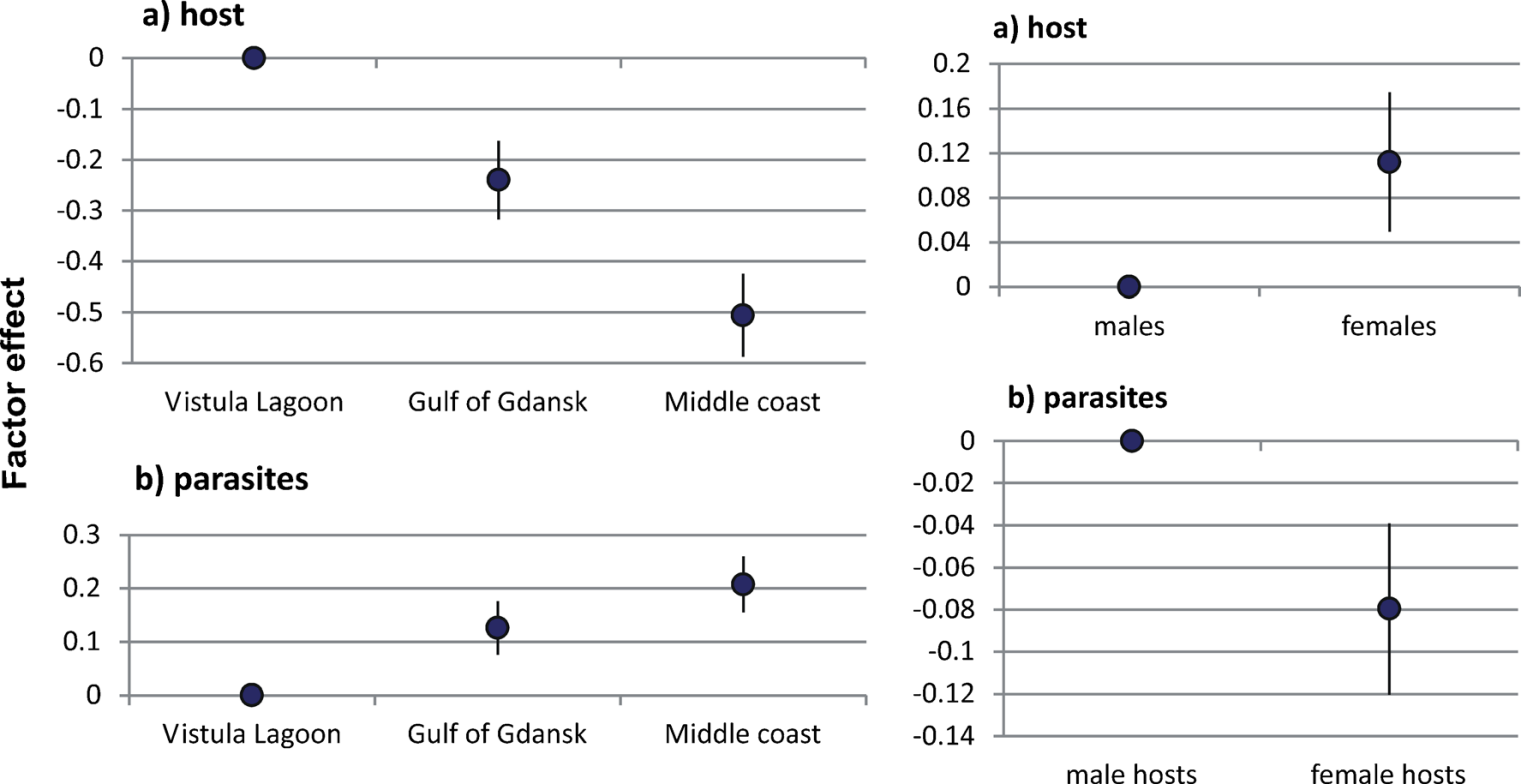
Effects of sampling area and sex of the host (with SE) estimated for models of AChE activity in herring muscle tissue (*host*) and *A. simplex* post-secretory larvae (*parasites*)

The GLM model of AChE activity in herring muscle tissue explained 27 % of the variance. The area effect was highest in the Vistula Lagoon and differed significantly from the Gulf of Gdansk (*p* = 0.003) and the middle coast (*p* < 0.001). The effect of fish sex was higher in females than in males, but the level of significance (*p* value) was only 0.075. The GLM model of AChE activity in *A. simplex* post-secretory larvae revealed the opposite biomarker response in parasites: the area effect was lowest in the Vistula Lagoon compared to the Gulf of Gdansk (*p* = 0.014) and the middle coast (*p* < 0.001). The effect of the host’s sex was significantly higher in parasites collected from male, as opposed to female hosts (*p* = 0.05). The model explained 13.5 % of the variance. The parameter estimates for the models of AChE activity in herring and *A. simplex* larvae are provided in Table 2. The AChE activity in the host–parasite system showed an inverse relationship (mirror effect) of area and sex effects (Fig. 3a, b).

**Table 2:** Parameter estimates (with SE) for models of AChE activity in herring muscle tissue (host) and *A. simplex* post-secretory larvae (parasites)

	Parameter/factor	Estimate	SE	*p*
Herring muscle tissue (host)	Intercept	2.85	0.07	<0.001
	Vistula Lagoon	0	Aliased	
	Gulf of Gdansk	−0.24	0.08	0.003
	Middle coast	−0.51	0.08	<0.001
	Males	0	Aliased	
	Females	0.11	0.06	0.075
*A. simplex* post-secretory larvae (parasites)	Intercept	4.74	0.04	<0.001
	Vistula Lagoon	0	Aliased	
	Gulf of Gdansk	0.13	0.05	0.014
	Middle coast	0.21	0.05	<0.001
	Male hosts	0	Aliased	
	Female hosts	−0.08	0.04	0.052

Variance accounted for in models: host 27 % and parasites 13.5 %

## Discussion

The data presented in this study show that mean AChE activity was approximately fourfold higher in E/S products and eightfold higher in somatic extracts of post-secretory *A. simplex* larvae than in the muscle tissue of its host, herring. Strikingly, the AChE activity level in nematodes was inversely related to the enzyme activity in their hosts: for example, reduced AChE activity in herring was accompanied by increased enzyme activity in its parasites. The threshold of sensitivity to insecticides is usually low in marine organisms, and exposure to pesticides at concentrations of 0.1–1 μg/l may lead to AChE inhibition in fish (Klaverkamp and Hobden 1980; Habig et al. 1986). Enzyme activity measurements revealed a 20 % depression of AChE activity in herring sampled in the Gulf of Gdansk, compared to herring in the Vistula Lagoon. Such level of reduction in AChE activity (20 %) in fish and invertebrates indicates exposure to neurotoxic compounds (Zinkl et al. 1987; Busby et al. 1989). In the middle coast samples, >40 % AChE inhibition in the host was observed, but enzyme activity was highest in parasites from this area. According to Dizer et al. (2001), depression of AChE activity by 20 to 50 % in marine organisms indicates sublethal impact of contamination with neurotoxic agents. These results are in line with our previous studies (Podolska and Napierska 2006) in which AChE inhibition in the muscle tissue of herring caught in contaminated areas was accompanied by very high enzyme activity in the body extracts of *A. simplex* larvae. It must be emphasized that presented results refer to enzyme activity in post-secretory *A. simplex* larvae, and AChE inhibition in herring sampled in middle coast was also accompanied by increasing enzyme activity in E/S products of nematodes.

*A. simplex* larvae have a high threshold of sensitivity to carbofuran (Podolska et al. 2008): no AChE inhibition was recorded in body extracts of larvae after exposure to carbofuran concentrations of up to 500 μg/l. Furthermore, the GLM model indicated that AChE activity was significantly elevated in larvae exposed to high concentrations of this carbamate pesticide. Recent results, obtained by native polyacrylamide gel electrophoresis of the somatic extracts and E/S products from live parasites, indicate the presence of two molecular forms of AChE in *A. simplex* larvae (Podolska et al. 2012). This latest study also revealed that AChE activity was higher in tissue homogenates prepared from *A. simplex* larvae frozen after the secretion of the enzyme than in larvae frozen immediately after collection; moreover, AChE in the E/S products of live parasites was characterized by a high level of activity. The results suggest that *A. simplex* larvae may have developed a mechanism of resistance to neurotoxic agents.

In many species of invertebrates, these resistance mechanisms are directly related to the activity of AChE. Chang and Opperman (1991) showed that class C AChEs from root-knot nematodes of the genus *Meloidogyne* are highly resistant to OPs and CBs. Kang et al. (2011b) proposed that soluble AChE plays an important role in the chemical defence system of *B. xylophilus* against various xenobiotics. Mutations in the AChE gene, responsible for AChE insensitivity to insecticides, have been identified in several species of arthropods, i.e. *Musca domestica* (Kozaki et al. 2001; Walsh et al. 2001), *Drosophila melanogaster* (Charpentier and Fournier 2001; Fournier 2005) and mosquitoes (Perera et al. 2008). Resistance to insecticides may also be enhanced by overproduction of AChE (Fournier et al. 1992; Charpentier and Fournier 2001). Thus, the high threshold of sensitivity to carbofuran in *A. simplex* larvae may be attributable either to a form of AChE that is resistant to inhibitors or to overexpression of the enzyme. A third possible adaptive mechanism, however, might be the secretion of AChE by these nematodes in response to adverse environmental conditions. In this model, secreted AChE acts as a decoy molecule, which binds inhibitors and thereby reduces the exposure of neuromuscular AChE to neurotoxic agents.

Secreted AChEs perform functions that are non-overlapping with those expressed in the nematode neuromuscular system (Selkirk et al. 2005a) and are important in host–parasite interactions in many nematode species. Numerous roles for this form of the enzyme have been suggested, i.e. the modulation of intestinal peristalsis (Lee 1970) and the immune response of the host (Pritchard 1995), and even protection against enzyme inhibitors present in the host’s diet, which may lead to expulsion of the parasites by muscular contractions of the host (Selkirk et al. 2005b). An inverse relationship between enzyme activities in the host–parasite system is consistent with the hypothesis that these parasites enhance AChE secretion under the influence of neurotoxic compounds.

Alternatively, AChE secretion by parasites might not relate directly to anticholinergic poisoning, but instead be an indirect response to the effect of AChE inhibition in their host. Yet, another possibility is that *A. simplex* larvae produce a large amount of AChE to prevent the accumulation of host acetylcholine in the vicinity of the parasite. Bhattacharya et al. (1997) proposed that *Wuchereria bancrofti* microfilariae secrete AChE into the circulation to degrade host acetylcholine (ACh). Since ACh stimulates the release of lysosomal enzymes and phagocytosis, the immune response of the host is suppressed during infection. Accordingly, Hussein et al. (1999b) suggested that AChE secretion by nematode parasites should inhibit host secretory responses.

The relationship between the sex of the host and nematode AChE activity is difficult to explain. AChE activity levels were significantly higher in *A. simplex* larval extracts obtained from male, as opposed to female, herring (Podolska and Napierska 2006; Podolska et al. 2008). Similarly, sex-dependent differences in mean AChE activities were also reported for both post-secretory larvae and their E/S products (Podolska et al. 2012). The results presented in the current report are in accordance with earlier findings. The greatest divergence was recorded between AChE activity in male herring (lowest level) and post-secretory larvae (highest level): in this, the most extreme case, the enzyme activity was over 12 times higher in parasites than in their hosts. In addition, the effect of sex was significant in both GLM models (for herring and *A. simplex*) and shows an inverse relationship between host and parasite AChE activities. How the sex of the host influences AChE activity in its parasites is unclear, however, although we might speculate that host sex steroid hormones affect nematode AChE activity and/or the rate of enzyme secretion. On the other hand, it is possible that the sex of the host indirectly influences on the response of larvae; thus, AChE secretion may be induced by the low level of enzyme activity in male hosts.

In summary, AChE secretion by A*. simplex* may constitute an adaptive mechanism that promotes survival under adverse environmental conditions. Larvae probably increase secretion of this enzyme in response to a direct and/or indirect effect of neurotoxic compounds. This is the first report of such a phenomenon in *A. simplex*.
